# Empirical relationships between algorithmic SDA-M-based memory assessments and human errors in manual assembly tasks

**DOI:** 10.1038/s41598-021-88921-1

**Published:** 2021-05-04

**Authors:** Benjamin Strenge, Thomas Schack

**Affiliations:** Center for Cognitive Interaction Technology (CITEC), Neurocognition and Action Group, Bielefeld University, 33615 Bielefeld, Germany

**Keywords:** Computational models, Data processing, Software, Psychology, Human behaviour, Computational science

## Abstract

The majority of manufacturing tasks are still performed by human workers, and this will probably continue to be the case in many industry 4.0 settings that aim at highly customized products and small lot sizes. Technical systems could assist on-the-job training and execution of these manual assembly processes, using augmented reality and other means, by properly treating and supporting workers’ cognitive resources. Recent algorithmic advancements automatized the assessment of task-related mental representation structures based on SDA-M, which enables technical systems to anticipate mistakes and provide corresponding user-specific assistance. Two studies have empirically investigated the relations between algorithmic assessments of individual memory structures and the occurrences of human errors in different assembly tasks. Hereby theoretical assumptions of the automatized SDA-M assessment approaches were deliberately violated in realistic ways to evaluate the practical applicability of these approaches. Substantial but imperfect correspondences were found between task-related mental representation structures and actual performances with sensitivity and specificity values ranging from 0.63 to 0.72, accompanied by prediction accuracies that were highly significant above chance level.

## Introduction

Manual assembly by human workers still plays a crucial role in many industrial areas in the early 2020s and will likely continue to do so for many years to come. On the one hand, technical systems such as robots powered by sophisticated sensors and highly precise actuators become capable of performing more and more assembly actions autonomously. On the other hand, trends towards increased customization of products and correspondingly smaller lot sizes demand increasingly high flexibility. Humans stand heads and shoulders above machines in this regard despite impressive advancements in the field of machine learning and other artificial intelligence techniques. Unsurprisingly, the vast majority (72%) of manufacturing tasks were still performed by humans according to a recent survey report from Kearney^1^. Especially the automotive industry reportedly learned from a range of recent experiences that human workers had to be brought back to the production lines. In 2016, Markus Schaefer, head of production at Mercedes Benz, stated *“Robots can’t deal with the degree of individualization and the many variants that we have today”*, so the company was *“moving away from trying to maximize automation with people taking a bigger part in industrial processes again”*^2^. Japanese car manufacturer Toyota already initiated a similar re-introduction of manual labor a few years earlier^3^. On 13 April 2018, Tesla CEO Elon Musk^4^ tweeted that *“excessive automation at Tesla was a mistake”* and *“humans are underrated”*. Siemens CEO Joe Kaeser and management consulting firm Oliver Wyman therefore concordantly prognosticated that robots would not replace human workers in manufacturing anytime soon^5,6^.

Working on this premise, a large body of current research is concerned with building technical systems using augmented reality setups and other advanced technologies to assist human workers in learning and executing manual assembly tasks^7–18^. Ideally, such systems should show as little unneeded information as possible in order to save their human users’ attentional resources but provide helpful information when the worker would not know what to do, prevent them from doing something wrong, and support learning processes. These requirements make manual assembly processes interesting application scenarios for task-related human memory analyses based on the “structural-dimensional analysis of mental representations” (SDA-M) method^19^ and especially its recent extension by algorithmic approaches for automatized human error prediction^20^.

SDA-M involves a semi-automatized survey and calculation procedure that yields user-specific data about the strength of associations between mental representations of actions in the context of a specific overarching activity. This information about the underlying cognitive-perceptual action system can then be used to address individual needs^21^. Numerous studies have already used SDA-M to analyze a broad and diverse range of activities in the fields of manual action, sports and exercise^22–27^, rehabilitation^28,29^, and work processes^30^. Previous research has shown that SDA-M can retrieve and visualize mental representation structures in a way that it is interpretable by specifically trained scholars (e.g., with a background in psychology, sports science, or mathematics) and domain experts to identify individual issues related to action execution, which facilitates appropriate training for improving performance^21,31–35^. For example, mental representations related to gymnastics skills of novices and experts have been retrieved and assessed using SDA-M. Individual interventions based on this information reportedly accelerated and optimized the learning process to bring novices’ mental representation structures closer to those of experts^26,36,37^. Recently, it has been shown that SDA-M data can be automatically analyzed in order to assess the likelihood that a specific user would know which actions should be executed in a given situation during the activity. Three different algorithmic approaches, the *Analysis of Most-Probable Actions* (AMPA) and the *Correct Action Selection Probability Analysis* (CASPA) using either a default value (${\text {CASPA}}_d$) or an informed value as a decision threshold (${\text {CASPA}}_i$), have been shown to be highly consistent with the conventional SDA-M approach that involves manual assessment of SDA-M data visualizations (dendrograms) and related statistical parameters by specially trained human experts^20^. Is has also been demonstrated that AMPA and CASPA could generate individualized performance predictions for an action sequence from a predefined, choreographed movement pattern in karate (the *Kanku-dai kata*), which correlated significantly with practitioners’ formal expertise and with the likelihood of mistakes in action execution^38^. AMPA and CASPA are based on different computational cognitive models but share a common set of assumptions^20^.

This article reports on two studies that empirically investigated the practical relations between outcomes of SDA-M-based analyses and the occurrences of human errors in manual assembly. The studies were designed in an application-oriented way and therefore entailed realistic violations of several theoretical assumptions of the SDA-M-based assessment algorithms.

## Overview of the SDA-M method

SDA-M consists of several survey and analysis steps^19,20^ that are briefly summarized in the following paragraphs:

### Task analysis

First, the activity to be analyzed (here: the manual assembly task) is split into so-called “basic action concepts” (BACs)^19^, which represent an atomic action step, typically using an image and corresponding textual description. This is commonly done by the investigators (or assistance system designers) together with domain experts and practitioners of different expertise levels in order to establish a plausible and workable set of BACs.

### Split procedure and distance scaling

Next, these predefined action items (BACs) are shown to study participants (or workers) during the so-called “split procedure” using specialized software such as the *QSplit* SDA-M tool (available from Thomas Schack upon request for research purposes). All *n* actions (BACs) are shown in random order, and all $n-1$ other actions are compared to them (also in random order). For each pair of actions the participant (or worker) must decide whether they are directly associated during execution of the analyzed activity (e.g., assembly task). Based on these decisions, the software calculates correlation and distance values between each pair of actions. These values can be used as a metric for clustering and visualizing the actions (BACs) or for automatized algorithmic assessments.

### Hierarchical clustering and visualization

The SDA-M software can visualize a hierarchical agglomerative average-linkage clustering of the actions with dendrograms to facilitate the assessment of participants’ individual mental representation structures through human experts. Most of the aforementioned previous SDA-M applications utilized this approach. Further steps are possible to investigate feature dimensions or to determine the similarity between the mental representation structures for different participants or groups^19^.

### Automatized algorithmic assessment of SDA-M data

The *Analysis of Most Probable Actions* (AMPA) and *Correct Action Selection Probability Analysis* (CASPA) are different algorithmic approaches for predicting human error based on SDA-M data^20^. They automatize the assessment of memory structures to predict probable errors in action sequences.

Roughly, AMPA determines for each step of an action sequence whether the set of actions with minimal distance to the previously executed action (as retrieved by SDA-M) contains a correct follow-up action. This outputs a simple binary assessment for each action that indicates whether participants are expected to be able to choose a correct action as the next step.

The CASPA algorithm is derived from computational approaches of the cognitive architecture “Adaptive control of thought—rational” (ACT-R)^39,40^. It outputs a continuous measure for estimating the probability of correct action selection after each action step. Arbitrary thresholds for this probability can be used to determine whether assistance should be provided. This binarized output of CASPA is referred to as ${\text {CASPA}}_d$ when using a default threshold of 0.5, or as ${\text {CASPA}}_i$ when using an empirically informed task-specific threshold^20^.

## Research questions and study design overview

In practical applications some of the assumptions set for automatized analyses of task-related mental representation structures using the SDA-M-based AMPA or CASPA algorithms will commonly be violated to some degree. To address this, the actual accuracy of AMPA’s and CASPA’s predictions in manual assembly has been empirically investigated for two different tasks.

**Figure 1:**
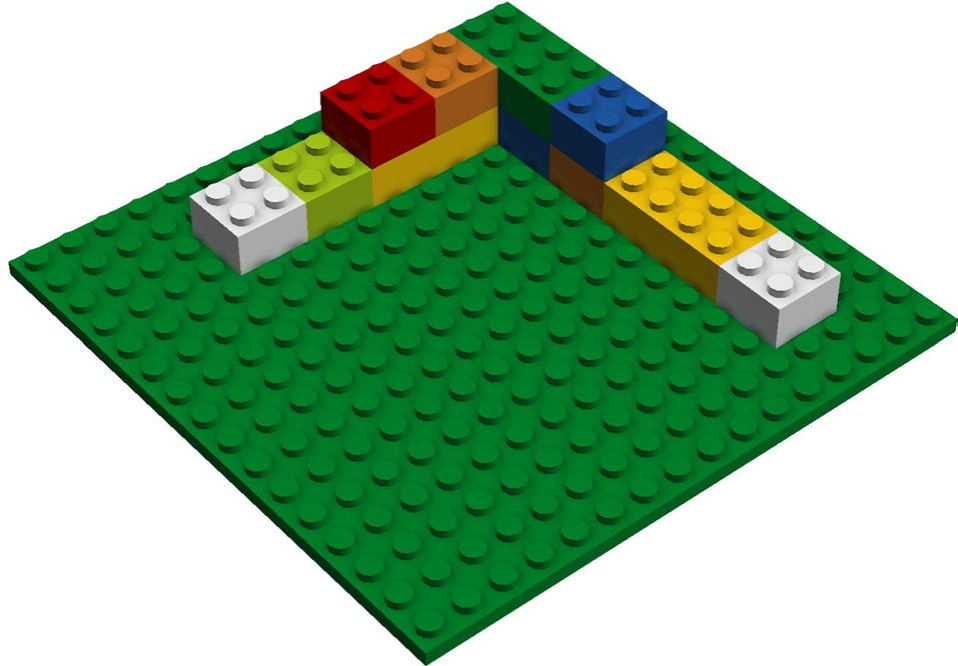
Duplo construction consisting of the first 12 parts of Funk’s standardized assembly task^8^.

**Figure 2:**
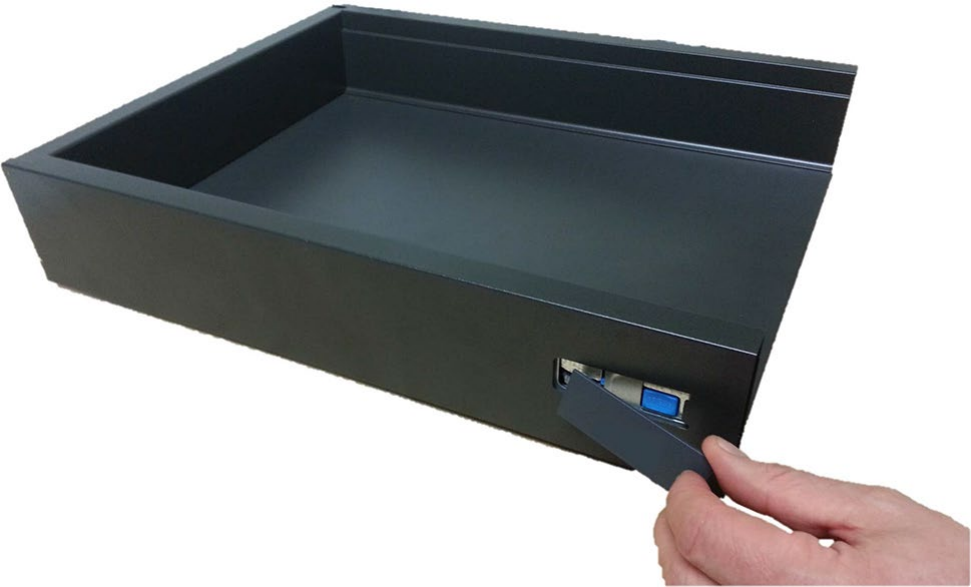
Drawer system mockup.

1. A cheap and easily reproducible pick-and-place assembly task derived from a standardized benchmark task^8^, which uses Lego Duplo bricks (see Fig. 1), and
2. a real-world assembly task from an industrial setting, which uses parts of a drawer system mockup by company Hettich (see Fig. 2).

This combination of tasks was chosen due to experimental feasibility, practical relevance, and so that different assumptions of AMPA and CASPA were violated to varying degree. Table 1 provides a rough overview how severely each assumption was violated in the two scenarios according to a 4-point ordinal scale from *“Not violated”*, *“Not substantially violated”*, and *“Moderately violated”*, up to *“Strongly violated”*. The first three assumptions (“atomicity”, “sequential discreteness”, and “non-recurrence”) could be satisfied by proper preparation and application of the SDA-M procedure, as is commonly the case for these assumptions. The remaining ordinal-scaled ratings were defined by the experimenters based on the following considerations:

- How many actions *not* included in the corresponding SDA-M split procedures could possibly (within a reasonably rational scope) be executed in each task scenario? (“Completeness”)
- How unremarkable, i.e., quiet, stable and non-distracting, is the test environment? (“Context-independence”)
- How much time passed between the split procedure and the task execution? (“Currentness”)

For example, in the Lego Duplo task it was mechanically possible to place any of the available differently sized and colored bricks anywhere on the green base plate or partial construction (see Fig. 1), which resulted in an unmanageable amount of possible actions that necessitated a strong violation of the “completeness” assumption to apply the SDA-M split procedure. The assembly parts in the Hettich study offered somewhat clearer affordances regarding their potential application and placement, thus limiting the action space.

**Table 1:** Degrees of violation of SDA-M algorithms’ assumptions in assembly studies.

Assumptions^20^	Duplo study	Hettich study
Atomicity	Not violated	Not violated
Sequential discreteness	Not violated	Not violated
Non-recurrence	Not violated	Not violated
Completeness	Strongly violated	Moderately violated
Context-independence	Not substantially violated	Strongly violated
Currentness	Not substantially violated	Not substantially violated

The two scenarios were examined independently from each other as discrete studies with disjunct groups of participants but using similar study designs. As a foundation for both studies, participants underwent a limited phase of learning or education about the assembly proceedings. Next, their task-related mental representation structures were retrieved with SDA-M software. Finally, they were asked to execute the assembly procedures. Any errors that were made during the assembly tasks were recorded and afterwards compared to the errors that the AMPA and CASPA algorithms would have predicted based on participants’ individual SDA-M data. The main differences between the two studies were related to the types of assembly actions, participants’ task-related education, and the control of contextual influences.

The Duplo assembly study used an experimental design with random assignment of participants to two groups. These groups received different material during an initial learning phase to induce heterogeneity concerning their task-related knowledge. While one half of participants received a printout with completely correct instructions for assembling the designated brick construction, the other half received instructions that contained some wrong assembly steps. These erroneous instructions were meant to simulate situations in which either the available blueprints or engineering drawings for a specific construction contain minor errors, or workers engage in building a new variant of a similar but slightly different construction they had learned to assemble in the past. The Duplo study was conducted in a quiet and controlled lab environment. In this study, a measurement of task-related memory structures with SDA-M was not only done *after* the learning phase (as in the Hettich study) but additionally also at the very beginning *before* the learning phase, in order to further validate the SDA-M-based assessment procedure. Since the assembly task was unknown to participants by then, a valid assessment of their task-related memory structures at this point was expected to be characterized by low probabilities of correct action selections, and differ from the corresponding assessment after learning.

The Hettich drawer assembly study used a quasi-experimental design that distinguished between participants with either more or less extensive task-related expertise (“experts” and “laypersons”). All participants were employees of company Hettich, one of the world’s leading manufacturers of furniture fittings. The experts group consisted of carpenters, joiners, and other workers with extensive task-related knowledge. The laypersons group consisted mainly of clerks, managers, and other office workers with limited professional experience in manual assembly. The study was conducted within an actual working environment at company Hettich in order to establish realistic conditions for practical assessment.

In summary, the research questions led to the following main hypothesis

- $H_1$ Algorithmic assessments of memory structures based on SDA-M data related to a specific assembly task correspond to subsequent outcomes of attempted action executions (success or error) in the respective assembly task,

and these supplementary hypotheses:

- $H_2$ *(Duplo study)* Algorithmic assessments of initial memory structures before learning of an unknown assembly task indicate a lack of task-related knowledge.
- $H_3$ *(Duplo study)* Algorithmic assessments of initial memory structures before learning of an unknown assembly task differ from the assessment of memory structures that are retrieved after learning.
- $H_4$ *(Hettich study)* The accuracy of algorithmic assessments of individual memory structures is independent of task-related expertise, i.e., the accuracy of prediction for “laypersons” does not differ from the accuracy of prediction for “experts”.

## Results

**Figure 3:**
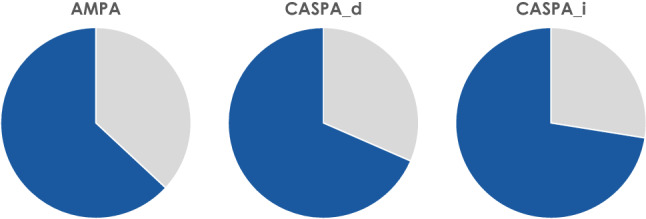
Sensitivity of SDA-M-based error prediction in assembly. Blue areas indicate the percentage of actual errors that could be correctly detected with AMPA (63%), ${\text {CASPA}}_d$ (68%), and ${\text {CASPA}}_i$ (72%) based on individual SDA-M data in two assembly scenarios.

All four complementary hypotheses could be supported by empiric evidence^41^. As a primary meta result of both studies, the SDA-M-based CASPA algorithm correctly predicted 68.5–72.5% of all errors and failures in manual assembly actions, depending on its threshold setting (see Fig. 3). The subsequent sections first describe additional consolidated results from both assembly studies combined (weighted by the respective numbers of trials; see also Table 2), and then the discrete results and ancillary findings for each study individually.

**Table 2:** Overall results of SDA-M-based error prediction in assembly.

Algorithm	Accuracy	Sensitivity	Specificity	PPV	NPV	Balanced accuracy
AMPA	$$0.68^{***,(\text{a})}$$	0.63	0.69	0.37	0.87	0.66
$${\text {CASPA}}_d$$	$$0.66^{***, (\text{b})}$$	0.68	0.65	0.36	0.88	0.67
$${\text {CASPA}}_i$$	$$0.65^{***, (\text{c})}$$	0.72	0.63	0.35	0.89	0.68

^(a)^$$p < 10^{-20}.$$

^(b)^$$p < 10^{-15}.$$

^(c)^$$p < 10^{-14}.$$

### Consolidated overall results

The overall accuracy, balanced accuracy^42^, and specificity values were comparable for all algorithmic variants (62.6–69.4%). One-tailed binomial tests with $H_0: P(\text {correctPrediction})\le {\frac{1}{2}}$ corroborated that the accuracies of all algorithms were highly significant above chance level (all $p < 10^{-14}$), providing support for the main hypothesis $H_1$.

Positive predictive values between 35.4 and 36.9% resulting from a relatively low prevalence of errors (149 errors in a total of 676 actions $\Rightarrow P({\text {error}}) \approx 22 \%$) indicated a notable chance of false alarms, but when the algorithmic SDA-M assessments predicted that an action would be correctly performed without assistance, this was correct in most cases (86.9–88.9%). Differences between the three algorithmic variants were marginal. Descriptively, AMPA had slightly higher specificity, whereas both versions of CASPA scored better regarding their sensitivity and negative predictive values.

### Detailed study-specific results

The results from both individual studies were similar to the consolidated overall values. In both studies and with all three variants of algorithmic assessments the match between SDA-M-based predictions and actual observations was significantly better than would be expected by chance (see accuracy and *p*-values in Tables 3 and 4).

**Table 3:** Results of SDA-M-based error prediction for Lego Duplo assembly.

Algorithm	Accuracy	Sensitivity	Specificity	PPV	NPV	Balanced accuracy
AMPA	$$0.71^{***, (\text{a})}$$	0.64	0.75	0.51	0.83	0.69
$${\text {CASPA}}_d$$	$$0.70^{***, (\text{b})}$$	0.69	0.70	0.49	0.84	0.70
$${\text {CASPA}}_i$$	$$0.70^{***, (\text{c})}$$	0.74	0.68	0.50	0.86	0.71

^(a)^$$p < 10^{-17}.$$

^(b)^$$p < 10^{-15}.$$

^(c)^$$p < 10^{-15}.$$

**Table 4:** Results of SDA-M-based error prediction for Hettich drawer assembly.

Algorithm	Accuracy	Sensitivity	Specificity	PPV	NPV	Balanced accuracy
AMPA	$$0.63^{***, (\text{a})}$$	0.59	0.64	0.17	0.92	0.62
$${\text {CASPA}}_d$$	$$0.60^{***, (\text{b})}$$	0.66	0.59	0.17	0.93	0.62
$${\text {CASPA}}_i$$	$$0.57^{**, (\text{c})}$$	0.66	0.56	0.16	0.93	0.61

^(a)^$$p < 10^{-5}.$$

^(b)^$$p = 0.0007.$$

^(c)^$$p = 0.0098.$$

Descriptively, for each of the three algorithms almost all metrics (except for negative predictive values) turned out slightly better in the Duplo assembly study than in the Hettich drawer scenario.

#### Auxiliary findings from Duplo study

The complementary analysis of participants’ initial task-related memory structures turned out as expected. CASPA estimated an average probability of only 18.7% that participants would have chosen correct actions for building the designated construction before they went through the learning phase for the Duplo assembly task, in contrast to a significantly higher assessed average probability of 57.8% after the learning phase (two-sided Wilcoxon signed-rank test, $W=2$, $p<10^{-10}$). This corroborates hypothesis $H_2$. While the assessments based on SDA-M measurements *after* the learning phase matched participants’ subsequent actual performance significantly *better* than would be expected by chance (see Table 3), the assessment of participants *initial* (pre-learning) memory structures matched their actual (post-learning) task performance significantly *worse* than flipping a coin (${\text {CASPA}}_d$, two-sided binomial test, $p=0.00016$). This corroborates the supposition that the automatized SDA-M-based assessments were actually sensitive to changes in participants’ task-related memory structures that were presumably caused by the learning phase, which is in line with hypothesis $H_3$.

**Figure 4:**
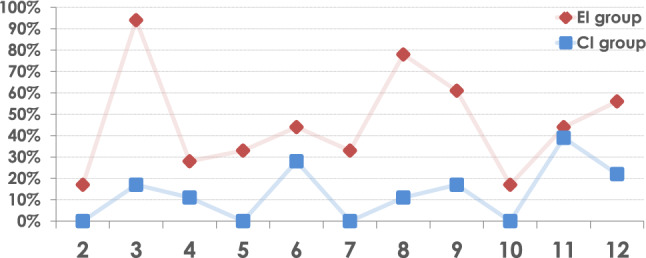
Frequencies of errors in each step of the Lego Duplo assembly task by participant groups.

As intended by the study design, participants in the Duplo study who received partially erroneous assembly instructions (“EI group”) made notably more errors than the correctly instructed participants (“CI group”). Unsurprisingly, the majority of participants in the EI group made mistakes in the three wrongly instructed assembly steps (see steps 3, 8, and 12 in Fig. 4). They also generally made significantly more errors than the CI group (91 errors vs. 26 errors in a total of 198 attempted action executions; two-sided Mann–Whitney test, $U=297.5$, $p=0.00001$), supposedly mainly due to increased levels of cognitive stress and confusion caused by the necessary corrective interventions after they made mistakes. These ancillary findings confirm that the study design worked as intended and successfully induced heterogeneity among participants concerning task-related knowledge and performance in order to yield more meaningful and robust main results.

#### Auxiliary findings from Hettich study

As a prerequisite for testing hypothesis $H_4$, the overall performances of laypersons and experts were compared to verify that participants were correctly assigned to the two groups. It could be confirmed that the experts were actually better at the tested assembly task: in total, the laypersons group made almost twice as many errors as the experts group (21 errors vs. 11 errors in a total of 140 attempted actions for each group). In this context it should be noted that some members of the experts group attempted to partially deviate from the officially defined reference procedure and choose alternative approaches for assembling the drawer. While these attempts were not actual errors in a narrower sense, the study design required them to be treated as such in order to enable proper comparisons. However, if these alternative approaches had been permitted, then the differences between laypersons’ and experts’ numbers of errors would have been even more pronounced in favor of the experts.

The accuracies of SDA-M-based assessments were calculated with all three algorithmic variants for each individual participant in both groups. Descriptive statistics (see Table 5) and the results of two-sided Mann-Whitney *U* tests did not indicate that the accuracies of SDA-M-based assessments differed between experts and laypersons for any of the three algorithms (AMPA: $U=94, p=0.87$; ${\text {CASPA}}_d$: $U=100.5, p=0.92$; ${\text {CASPA}}_i$: $U=93.5, p=0.85$). This corroborates hypothesis $H_4$.

**Table 5:** Central tendencies of individual assessment accuracies for Hettich drawer assembly by participant groups.

	AMPA		${\text {CASPA}}_d$		${\text {CASPA}}_i$	
	Mean (%)	Median (%)	Mean (%)	Median (%)	Mean (%)	Median (%)
Experts	65.0	60	61.4	60	57.9	55
Laypersons	61.4	60	57.9	60	56.4	60

## Discussion

Substantial connections were found between task-related mental representation structures and actual performances in manual assembly tasks. Overall, the empiric results concerning the relationship between automatized analysis of mental representation structures and action execution in the area of manual assembly were largely consistent with those recently found regarding sequential movements in sport^38^. In both conducted assembly studies the majority of human errors, as well as correct action selections, could be properly predicted with computational analyses based on participants’ individual SDA-M data. The overall low prevalence of errors led to comparatively low positive predictive values though. In order to take such uncertainties into consideration and make proper use of the available information, cognitive assistance systems could derive user-specific “configuration presets” from the SDA-M-based analysis results but enable proficient users to effortlessly discard unneeded suggestions and other proactively offered assistance features (in case of falsely predicted “errors”) in addition to offering means for (re-)activating wanted but absent assistance at all times.

The studies did not indicate a clear superiority of one algorithmic approach over another. All tested variants (AMPA, ${\text {CASPA}}_d$, and ${\text {CASPA}}_i$), which were based on different cognitive models and parameters concerning action selection mechanisms^20^, performed comparably well. For most practical applications in industrial assembly scenarios, sensitivity would likely be considered the most important metric in order to anticipate as many actual errors as possible. In this regard, the CASPA approach tended to work slightly better than AMPA. The ${\text {CASPA}}_i$ algorithm would have correctly predicted approximately eight out of eleven actual errors in the studies, but the remaining three errors would have been unanticipated. For this reason, practical applications cannot rely solely on this information as a means for preventing all possible errors. This was not surprising from a theoretical point of view for two reasons: first, the assumption of completeness was violated by restricting the number of possible actions that were considered in the SDA-M split procedures. Since split procedures have a time complexity of $\Theta (n^2)$, i.e., the time for completing them grows quadratically as a function of the number of actions, limiting the cardinality of action sets is essential for all practical applications. Second, the task-related mental representation structures retrieved by SDA-M can generally only indicate individual mistakes but not situation- or context-related slips that may arise e.g., due to temporary distractions. For this reason it is also not surprising that the tightly controlled lab study (Duplo assembly) descriptively indicated slightly stronger relationships between assessments of mental representation structures and actual performances than the data acquired in the more unstable surroundings that served as a realistic test environment at company Hettich. Note that, since the two studies differed not only in terms of environmental controlledness but also several other aspects, a direct comparison for drawing conclusions in this regard is not feasible yet and requires further research that systematically focuses on this aspect.

Overall, the results make automatized SDA-M-based assessments of workers’ task-related memory structures in manual assembly appear promising as a means for providing individualized on-the-job training or tailoring cognitive assistance systems to users’ personal requirements. However, we expect that the actual real-life usefulness of these approaches will largely depend on specific properties and requirements of the intended application context. Future long-term studies should investigate this further. The consolidated meta results presented in this research article were based on data from two very different assembly tasks involving a diverse sample of participants in terms of age, sex, educational background, and task-related experience, in order to enhance the robustness and generalizability of the results. However, even if the outcomes of the Duplo assembly and Hettich drawer assembly studies were roughly comparable, it cannot be ruled out that substantially different results would be found in other assembly scenarios and with workers that have other cognitive characteristics than the samples in these studies. Notable limitations of the studies include that factors such as the duration of learning, the time elapsed between learning and task execution, and the number of required assembly actions did not substantially vary within the studies. Future studies could systematically manipulate these factors as independent variables to assess their impact. Another worthwhile goal for further research would be to investigate the stability or volatility of task-related mental representation structures and corresponding assessments over time depending on the frequency of task executions. Generally, violations of the currentness assumption are expected to decrease prediction quality to an as yet unknown degree. Ideally, this could yield practically useful insights about how frequently the SDA-M split procedure needs to be repeated and how to adequately track workers’ learning curves.

## Methods

### Statement of ethical approval

Both studies have been approved by the ethics committee of Bielefeld University in written form according to the guidelines of the German Psychological Society (DGPs) and the Association of German Professional Psychologists (BDP). All participants gave informed and written consent to participate in the study.

### Participants

#### Duplo study

$N_{\text {D}}=36$ individuals between 18 and 38 years with a mean age of 24.5 years ($SD=4.3$) participated in the study. The acquisition was based on a call for participation in the form of textual announcements placed on several walls of Bielefeld University and the FH Bielefeld University of Applied Sciences. Therefore it is safe to assume that most participants were students or employees of these universities. They were either reimbursed for their time with 5 Euros in cash, or credited with one hour of experimental participation in partial fulfillment of the requirements of an eligible study program at Bielefeld University. The majority (83%) of participants were female. One half of all participants was randomly assigned to the CI group and the other half to the EI group.

#### Hettich study

$N_{\text {H}}=28$ individuals between 23 and 59 years with a mean age of 40 years ($SD=10.2$) participated in the study. All participants were employees of company Hettich who had been recruited by our contacts and asked to participate on their own volition during their working hours. The majority (75%) of participants were male. Our contacts used their personal knowledge and informed judgment to assign 50% of all participants to the “laypersons” group and the other half to the “experts” group for the assembly task.

### Duplo study procedure

**Figure 5:**
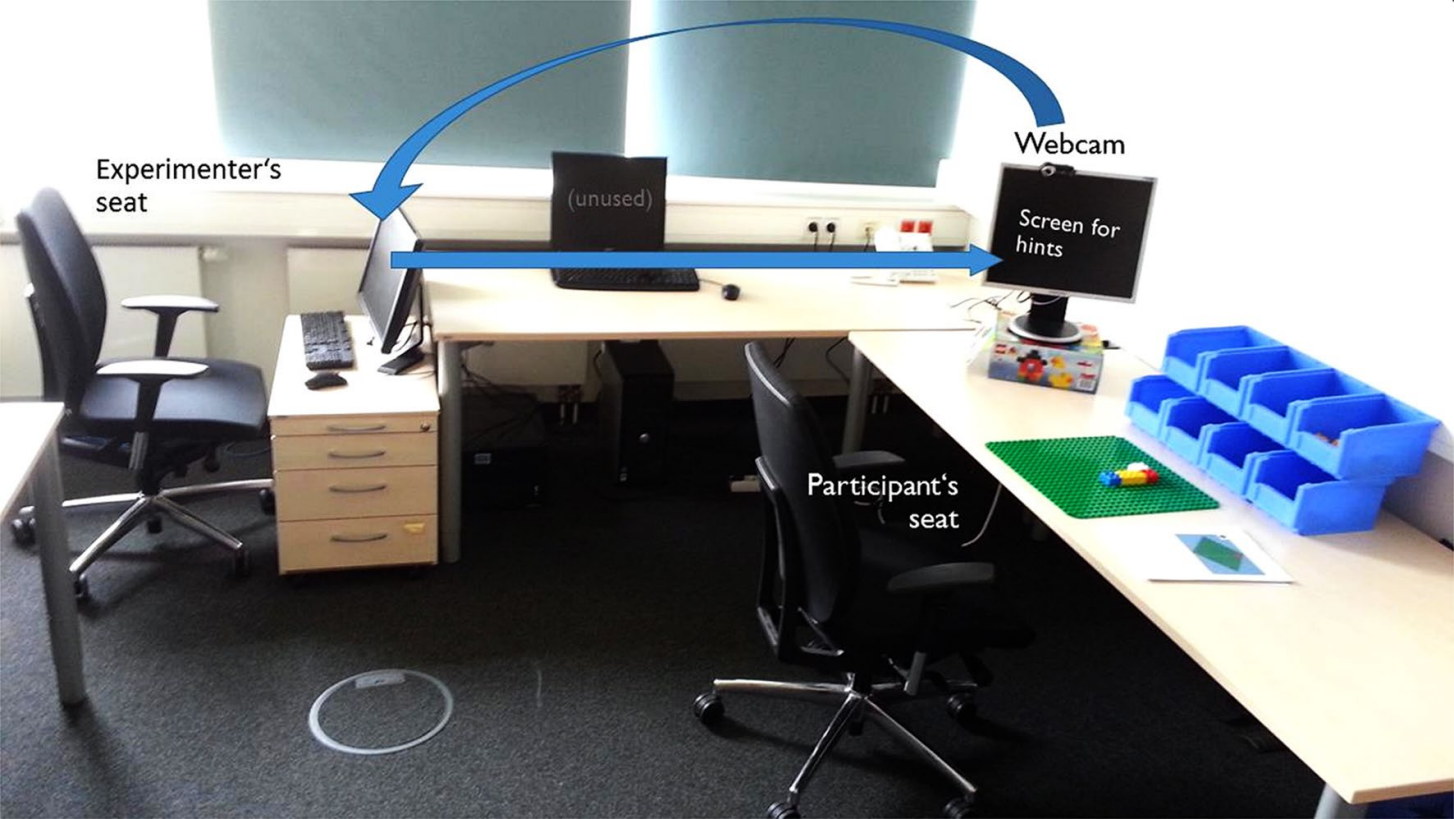
Lab setup for the Duplo assembly study. A webcam live stream of the assembly area enabled the experimenter to observe participants’ actions and intervene on errors by triggering an auditory signal and display the correct action on a screen next to the assembly area.

First, participants were welcomed and asked to fill out a questionnaire with demographic data and an informed consent for participation, including audio/video recording of their trial. The physical lab setup is shown in Fig. 5. Participants were seated in front of a table with a large green Lego Duplo base plate and eight blue boxes that each containing a specific type of Lego Duplo brick with a unique combination of size and color. A computer screen with webcam was placed to their left. The blue boxes were covered by a blanket throughout the experiment except when participants actually needed to use them. During assembly, the webcam recorded the task execution and streamed a live image to the experimenter’s screen in the back. This arrangement ensured that participants could not see the experimenter who silently observed their actions during the trial in order to mitigate potential experimenter effects. When assembly errors occurred, the experimenter used the computer to intervene by sending hints to the participants’ screen. The procedure of each experimental trial was divided into four steps: SDA-M introduction and pretest, task-related learning phase, SDA-M posttest, and self-reliant task execution. These are subsequently described in more detail.

#### SDA-M introduction and pretest

Participants were instructed how to make decisions during the SDA-M split procedure. Depending on participants’ native language, the split instruction either read

*“Are the depicted steps sequentially associated during assembly, i.e. performed immediately before/after another during task execution?”* (English)

or

*“Sind die dargestellten Aktionsschritte sequentiell zusammengehörig, d.h. werden sie unmittelbar vor-bzw. nacheinander durchgeführt?”* (German)

Printed examples of some assembly actions (with respect to a hypothetical Duplo construction not used in the actual study afterwards) and related SDA-M split decisions (marked as correct or incorrect) were handed out to participants. When they had worked through the examples, three test cases were shown and participants were asked for their decision to verify that they had understood the instructions.

**Figure 6:**
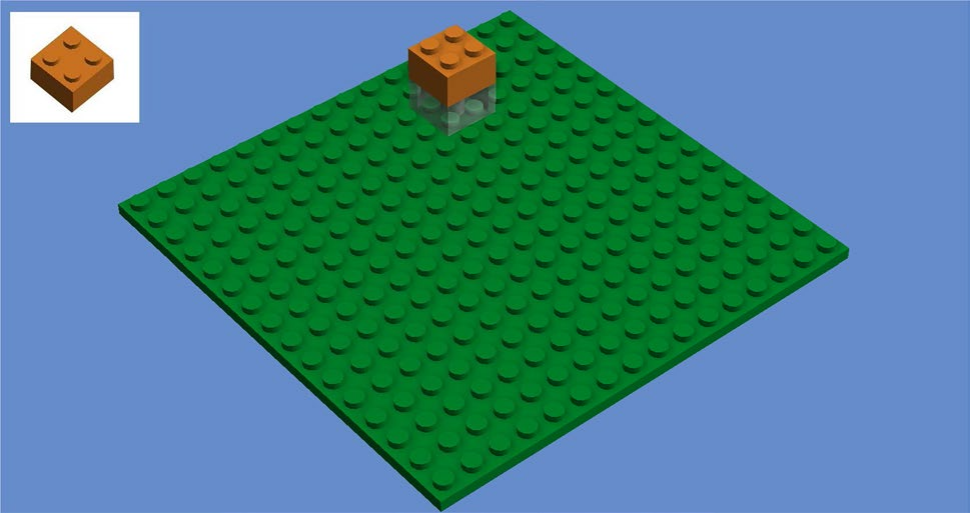
Pictorial representation of a placement action in the Duplo assembly study. The transparent placeholder brick indicated that the new orange brick must be added on top of another brick at the same X,Y position. The QSplit user interface for the SDA-M split procedure also showed a simple textual description of the action (*“Placing a small orange brick”*).

An SDA-M pretest using the QSplit SDA-M software on a tablet computer was then conducted in order to verify that algorithmic assessments of participants’ task-related memory structures reflected their lack of applicable previous knowledge regarding the task structure before they learned about it. A picture of the final result of task execution as shown in Fig. 1, i.e., the complete target construction consisting of the first 12 bricks from a standardized 16-brick construction^8^, was briefly shown to participants (for 1 s) prior to the SDA-M split procedure, so they could have recognized it if they had known it and were informed which activity the split procedure refers to. As expected, all participants later confirmed verbally that they did not recognize or know how to build the construction at this point. Each action representation in the SDA-M split procedure described a single assembly step, i.e., placing one brick. The images only displayed the new brick that was to be added in the respective step but not any other bricks that would already have been placed in previous steps (see Fig. 6 for an example). This simplified type of pictorial action representation was chosen because in most cases showing all previously placed bricks as well would have made it rather trivial to infer the sequential order of placement actions (and corresponding decisions in the split procedure) simply by checking whether the images differed by exactly one brick. In order to ensure consistency and comparability between the experimental phases and groups, action representations for the 12 correct assembly steps as well as for the three wrong actions from the EI group instructions have been incorporated in the SDA-M split procedure, resulting in a total of 15 action representations. This selection was in line with the prevailing approaches in the previous research, which either confined SDA-M split procedures exclusively to representations of actions that constitute correct action sequences for a given task^24,26,28,29,43^ or additionally included representations of a few typical errors^44^. Since in principle any kind of brick could have been placed anywhere on the base plate in any step, this confinement of the split procedure strongly violated AMPA’s and CASPA’s theoretical assumption of completeness.

#### Learning phase

The task then had to be learned by participants so that they would be able to execute it reasonably well. Since the task was previously unknown to participants, this learning phase was obviously necessary to activate or establish some basic task understanding, the related problem solving operators and meaningful task-related mental representation structures in the first place. The contents of the instructions represent an independent variable with two different levels: The CI group (task execution guided by fully correct pictorial step-by-step instructions) and the EI group (task execution guided by partially incorrect pictorial step-by-step instructions with “wrongly” colored bricks in assembly steps 3, 8, and 12). This learning material resembled the type of printed step-by-step assembly instructions used by Funk^8^ and was similar to the stimuli used to represent action steps in the split procedure (as in Fig. 6) but additionally contained all bricks from previous steps (i.e., the entire state of the construction at a specific instant). Participants were informed that only the relative positions of bricks were actually relevant, not the absolute position related to the green base plate. The learning phase was limited to 4 min. Participants could assemble and disassemble the construction and look at the step-by-step instructions as many times as they wanted within this time frame. After the learning phase, participants were instructed to turn away from the assembly area and let the experimenter disassemble whatever they built, replace all bricks to their respective boxes, and cover them with a blanket.

#### SDA-M posttest

Next, the SDA-M split procedure was performed again to update the data about participants’ task-related mental representation structures. A picture of the complete designated 12-brick reference construction was again briefly shown to participants (for 1 s) prior to the SDA-M split procedure.

#### Assembly task execution

Participants were then asked to execute the task (i.e., build the designated construction) without guidance, i.e., solely based on their own task knowledge. They were instructed to only touch those pieces they needed to assemble in the current step. All required bricks for the assembly task were arranged in the boxes on the table in front of them. The experimenter supervised the assembly by observing a live camera image. Whenever a participant put his or her hand in a box containing pieces that were not needed in the current step, as well as when they placed a correct brick at a wrong position, this counted as an error. Apart from that, errors were also counted when a participant claimed to not know how to proceed. Whenever a participant made such an error during action execution, the experimenter triggered an assisting hint for the participant which was announced by an audio signal and displayed the correct assembly action for 5 s on the participant’s screen to their left. This enabled participants to always continue with a correct subassembly at any point within the process.

### Hettich study procedure

This study took place in a spacious industrial working environment of company Hettich. Two trials were executed in parallel in different partitions of the hall, each by a dedicated experimenter. The space between the two assembly areas was large enough to prevent participants from directly and deliberately interacting, so they could not assist or copy from each other. However, indirect interference factors such as mutual distraction due to noises during assembly were deliberately left uncontrolled.

First, participants were welcomed and asked to fill out a questionnaire with demographic data and an informed consent for participation. The subsequent procedure of each experimental trial was divided into three steps: assembly-related instruction, SDA-M introduction and split procedure, and self-reliant task execution. These are subsequently described in more detail.

#### Assembly-related instruction

Participants received printed instructions with pictorial and textual descriptions how to assemble a specific drawer system mockup in eleven steps based on educational material from company Hettich. In order to account for participants’ considerably differing task-related capabilities and previous knowledge no strict time limit was imposed on the learning phase. Participants were asked to take reasonable time looking through and trying to remember the instructions, and inform the responsible experimenter when they felt ready.

#### SDA-M introduction and split procedure

The SDA-M split procedure was explained by showing participants a special tutorial video included in the QSplit software, which specifies the instructions as follows (in German):

*Die Software blendet Darstellungen von je zwei Teilschritten der Handlung ein. Sie sollen entscheiden, ob diese Teilschritte bei der Durchführung “direkt sequentiell zusammenhängen” oder nicht, d.h. ob diese unmittelbar vor-oder nacheinander durchgeführt werden. Hierbei spielt keine Rolle, welcher Teilschritt links bzw. rechts angezeigt wird.*

In English this translates to:

*The software shows representations of two action steps. You shall judge whether these action steps are sequentially “directly associated” during task execution or not, i.e., whether they are executed immediately before or after another. Hereby it does not matter which action step is shown on the left side and which one on the right side of the screen.*

The tutorial video continues to illustrate the implications of these instructions using a simple exemplary action sequence for toasting white bread slices and the respective decisions in a corresponding split procedure. Participants were asked to confirm whether had understood these general instructions.

After this, they were subjected to an SDA-M split procedure related to the drawer mockup assembly process. The split procedure incorporated pictorial and textual representations of all eleven assembly steps from the intended action sequence. As an example, Fig. 2 shows the pictorial representation of the final assembly action *“Fixate Hettich logo at the frame”*.

#### Assembly task execution

Lastly, participants were asked to assemble the Hettich drawer system mockup. All required parts and tools were previously placed on a work bench. An experimenter stood by and observed the assembly process. When participants attempted to execute any unintended actions the experimenter took note, intervened verbally by telling them to first reverse the wrong action (if applicable) and helped them execute the correct action instead. This enabled participants to always continue with a correct subassembly at any point within the process.

## Data Availability

The datasets generated during and/or analysed during the current study are available in the Bielefeld University PUB repository, https://doi.org/10.4119/unibi/2945514.
